# Can bacteria think?

**DOI:** 10.1038/s44319-024-00334-z

**Published:** 2024-11-25

**Authors:** Howy Jacobs

**Affiliations:** Tampere University, Tampere, Finland

**Keywords:** Evolution & Ecology, Microbiology, Virology & Host Pathogen Interaction

## Abstract

The mapping of the *Drosophila* neural connectome is a scientific tour de force. But could bacterial communication be just as sophisticated?

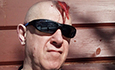

The landmark achievement of mapping the connectome of the *Drosophila* brain (Dorkenwald et al, 2024; Schlegel et al, 2024; Lin et al, 2024) opens up many new avenues for neuroscience. The fly brain contains more than 100,000 neurons, and their connections indicate the existence of thousands of individually classifiable cell-types. Made possible by FlyWire (https://flywire.ai/), a large international consortium, the project and its outcomes pave the way for understanding how metazoan brains, including our own, actually work.

Although I am not in any sense a neuroscientist, FlyWire’s impressive feat renews my own curiosity about other systems in nature that may be comparable in complexity, sophistication, and functionality. In particular, we should note that a single colony of *E. coli* can comprise a thousand-fold more individual cells than a fly brain. Our understanding of how bacterial cells communicate—and to what end—has advanced considerably in the past 50 years, notably with the elucidation of the mechanisms of chemotaxis, quorum sensing, and the manufacture of ‘secondary metabolites’ that we initially recognized as antibiotics. And this does not even include the ease with which bacteria constantly exchange genetic material among each other to quickly adapt to their environment. More recently we have come to recognize that the myriad of bacterial species, as well as archaea, that inhabit a given environment such as the soil, the ocean, or the mammalian gut, constitute a complex ecosystem that relies upon intricate intercellular communication for its ‘health’ and survival.

These aspects of microbial life have taught us that bacteria and archaea are not simply inert or passive organisms, but can play major roles in shaping the environments in which they live. But a persistent misconception about prokaryotes is that, as representatives of the most ancient cells, they are primitive in their biology, frozen into the same limited set of competencies that characterized their ancestors. Whilst this assertion might be true, I consider it inherently untrustworthy, since it is promoted by a sentient species that considers itself the pinnacle of evolution, arrogantly speaking on behalf of eukaryotes as a whole.

It is a truism about scientific discovery that we don’t know about the existence of something until it is discovered. After discovery it can sink back into relative obscurity, considered as something trivial and obvious. But just because we don’t yet know of its existence, nor even conceive of the physical mechanisms by which it could ever exist or function, does not mean that it is not of great importance in the universe. The nervous system of a fly would have been in this category 150 years ago.

Here I would like to advance the hypothesis—only a hypothesis because we do not yet have any substantive evidence for its existence— that bacterial communities, like metazoan neurons, operate an advanced system of intra- and inter-cellular communication with a computing power that challenges anything invented by humans or, indeed, by any eukaryote. My idea is not wholly original. The notion that bacteria can learn and evolve characteristics akin to intelligence was, for instance, recently supported by Rafael Lahoz-Beltra, a biomathematician based in Madrid, whose ideas are summarized in Lahoz-Beltra et al (2014). Many others have picked up the baton, for example, Somathilaka et al (2023), who demonstrated that the gene regulatory networks of bacteria have properties resembling those of a neural network, advancing the idea that this feature could one day be mobilized to develop non-silicon based computing.

Lehaz-Beltra’s group has developed the concept further by designing a perceptron (a supervised learning algorithm) that enables *E. coli* to optimize the production of a metabolite. They then engineered it in physical form into a plasmid for expression, using the basic toolkit of synthetic biology (Becerra et al, 2022). A key aspect of their thinking is that such decision-making is not the product of a single cell, but of a network of cells in communication via their environment; it remains to be confirmed whether the plasmid actually works in living cells as predicted.

These ideas are built on known and testable properties of proteins, metabolites, and nucleic acids. Moreover, they are, by definition, a usufruct: a construct that exploits the capabilities of one actor, in this instance bacteria, to achieve a goal designed by another, here a human. Whether *E. coli* can do it all naturally, by itself, remains an open question.

My own proposition, by contrast, belongs for now in the realm of science fiction. Its physical basis, if any, is unknown. Therefore its very existence is fanciful. But that does not rule out the possibility that it is real. If bacteria can learn, remember and solve problems this is only one step away from asserting that they can think.

If mere survival were considered the sole marker of evolutionary success, we would never have paid any attention to a Mozart, an Einstein, or an Elizabeth Taylor. But to graft onto bacteria this very human idea of what thought is all about is both arrogant and unwarranted. We can but note that the genetic tools that operate in the bacterial world, which we do know something of, do permit vastly accelerated, adaptive evolution compared with our own plodding reliance on sex.

Bacteria may be much smarter than we imagine, yet even this concept is anthropomorphic as well as speculative. Obviously, we ourselves still have much to learn.

## Supplementary information

Peer Review File

## References

[CR1] Becerra AG, Gutiérrez M, Lahoz-Beltra R (2022) Computing within bacteria: programming of bacterial behavior by means of a plasmid encoding a perceptron neural network. Biosystems 213:104608. 10.1016/j.biosystems.2022.104608 35063580 10.1016/j.biosystems.2022.104608

[CR2] Dorkenwald S, Matsliah A, Sterling AR, Schlegel P, Yu SC, McKellar CE, Lin A, Costa M, Eichler K, Yin Y et al (2024) Neuronal wiring diagram of an adult brain. Nature 634:124–138. 10.1038/s41586-024-07558-y 39358518 10.1038/s41586-024-07558-y PMC11446842

[CR3] Lahoz-Beltra R, Navarro J, Marijuán PC (2014) Bacterial computing: a form of natural computing and its applications. Front Microbiol 5:101. 10.3389/fmicb.2014.00101 24723912 10.3389/fmicb.2014.00101 PMC3971165

[CR4] Lin A, Yang R, Dorkenwald S, Matsliah A, Sterling AR, Schlegel P, Yu SC, McKellar CE, Costa M, Eichler K et al (2024) Network statistics of the whole-brain connectome of Drosophila. Nature 634:153–165. 10.1038/s41586-024-07968-y 39358527 10.1038/s41586-024-07968-y PMC11446825

[CR5] Schlegel P, Yin Y, Bates AS, Dorkenwald S, Eichler K, Brooks P, Han DS, Gkantia M, Dos Santos M, Munnelly EJ et al (2024) Whole-brain annotation and multi-connectome cell typing of Drosophila. Nature 634:139–152. 10.1038/s41586-024-07686-5 39358521 10.1038/s41586-024-07686-5 PMC11446831

[CR6] Somathilaka SS, Balasubramaniam S, Martins DP, Li X (2023) Revealing gene regulation-based neural network computing in bacteria. Biophys Rep 3:100118. 10.1016/j.bpr.2023.100118 10.1016/j.bpr.2023.100118 PMC10462848 37649578

