# Supplementary material for: Can bacteria think?
Source: EMBO Rep. 2024 Nov 25;26(1):3–4. doi: 10.1038/s44319-024-00334-z (PMC11723906; doi:10.1038/s44319-024-00334-z)
Supplement: Supplementary file 1 — Peer Review File [file 44319_2024_334_MOESM1_ESM.pdf]

EMBO reports

Can bacteria think?

Howard Jacobs

Corresponding author(s): Howard Jacobs (howard.jacobs@tuni.fi)

Review Timeline:

Submission Date:		15th Nov 24
Accepted:		18th Nov 24

Editor: Holger Breithaupt

Transaction Report: This article was editorially reviewed.
